# Dyslipidemia Increases the Risk of Incident Kidney Stone Disease in a Large Taiwanese Population Follow-Up Study

**DOI:** 10.3390/nu14071339

**Published:** 2022-03-23

**Authors:** Jia-An Hung, Chien-Hsun Li, Jiun-Hung Geng, Da-Wei Wu, Szu-Chia Chen

**Affiliations:** 1 Department of Post Baccalaureate Medicine, Kaohsiung Medical University, Kaohsiung 807, Taiwan; jackjean629@gmail.com; 2 Department of Neurology, Kaohsiung Medical University Hospital, Kaohsiung Medical University, Kaohsiung 807, Taiwan; gavli@kmu.edu.tw; 3 Department of Neurology, Kaohsiung Municipal Siaogang Hospital, Kaohsiung Medical University, Kaohsiung 812, Taiwan; 4 Integrated Center of Healthy and Long-Term Care, Kaohsiung Municipal Siaogang Hospital, Kaohsiung Medical University, Kaohsiung 812, Taiwan; 5 Department of Urology, Kaohsiung Municipal Siaogang Hospital, Kaohsiung Medical University, Kaohsiung 812, Taiwan; u9001090@gmail.com; 6 Department of Urology, Kaohsiung Medical University Hospital, Kaohsiung Medical University, Kaohsiung 807, Taiwan; 7 Department of Internal Medicine, Kaohsiung Municipal Siaogang Hospital, Kaohsiung Medical University, Kaohsiung 812, Taiwan; u8900030@yahoo.com.tw; 8 Department of Internal Medicine, Division of Pulmonary and Critical Care Medicine, Kaohsiung Medical University Hospital, Kaohsiung Medical University, Kaohsiung 807, Taiwan; 9 Department of Internal Medicine, Division of Nephrology, Kaohsiung Medical University Hospital, Kaohsiung Medical University, Kaohsiung 807, Taiwan; 10 Faculty of Medicine, College of Medicine, Kaohsiung Medical University, Kaohsiung 807, Taiwan; 11 Research Center for Environmental Medicine, Kaohsiung Medical University, Kaohsiung 807, Taiwan

**Keywords:** kidney stone disease, lipid profile, follow-up, Taiwan Biobank

## Abstract

The prevalence and incidence rates of kidney stone disease (KSD) in Taiwan are high; however, the association between lipid profile and KSD has yet to be investigated. The aim of this longitudinal study was to investigate the association between lipid profile with baseline and incident KSD in a large Taiwanese cohort. A total of 27,002 people enrolled in the Taiwan Biobank (TWB) were followed for a median of 4 years and classified into two groups according to whether they had (*n* = 1813; 6.7%) or did not have (*n* = 25,189; 93.3%) KSD at baseline. The presence of KSD was defined according to a self-reported history of kidney stones. The participants with baseline KSD (*n* = 1813) were excluded from the follow-up study, and the remaining participants were classified into two groups consisting of those who had (*n* = 640; 2.5%) or did not have (*n* = 24,549; 97.5%) incident KSD. After multivariable analysis, compared to quartile 1 of lipid profile, the participants in quartile 4 of triglycerides, quartiles 3 and 4 of high-density lipoprotein cholesterol (HDL-C), and quartile 4 of total cholesterol (Chol)/HDL-C ratio were significantly associated with baseline KSD. In the follow-up study, the participants in quartiles 2, 3, and 4 of triglycerides; quartile 2 of Chol; quartile 4 of HDL-C; quartile 3 of LDL-C; and quartiles 3 and 4 of Chol/HDL-C ratio were significantly associated with incident KSD. Our results showed that hypertriglyceridemia (67–93 mg/dL) was associated with a 1.463-fold increased risk of incident KSD and that low HDL-C (>63 mg/dL) protected against incident KSD formation. In addition, a Chol/HDL-C ratio larger than 3.64 was associated with a 1.381-fold increased risk of incident KSD. Our findings may imply that the optimal management of dyslipidemia may be associated with a lower risk of developing kidney stones.

## 1. Introduction

Kidney stone disease (KSD) is an increasing healthcare problem in Taiwan and worldwide [1]. In Taiwan, the overall age-adjusted prevalence rates of KSD are approximately 9%, 6%, and 7% in males, females, and all subjects, respectively [2]. KSD often recurs, with relapse rates of 50% in 5–10 years and 75% in 20 years [3]. The etiology of KSD is multifactorial and is associated with genetic predisposition, dietary habits, climate change, recurrent urinary infections, anatomical abnormalities, and medical conditions [4]. Among the medical conditions, metabolic syndrome has been associated with an increased risk of KSD [5,6,7,8,9,10]. Moreover, each component of metabolic syndrome including elevated body mass index (BMI) [9], dyslipidemia [11,12,13,14,15], diabetes [16,17], and hypertension [18,19] has also been associated with an increased risk of KSD. The complications of KSD include urinary obstruction, hydronephrosis, and pyelonephritis, which can lead to urosepsis, the leading cause of KSD-related mortality [20,21]. KSD is also associated with many comorbidities and increased risks of metabolic bone disease, chronic kidney disease, and cardiovascular events [22]. Hence, determining the risk factors for KSD is vital so that clinicians can optimally manage patients and prevent these complications.

Dyslipidemia is a common disorder globally. In Taiwan, the mean prevalence rates of hypertriglyceridemia and hypercholesterolemia are 29.26% and 43.96%, respectively, which are similar to the United States [23,24]. Dyslipidemia is a well-established risk factor for cardiovascular diseases, including cerebrovascular accident and coronary heart disease [25,26]. A study regarding the association between dyslipidemia and KSD reported a higher prevalence of dyslipidemia in patients with stone formation compared to those without stones [11]. Another study suggested a weak association [27], whereas another study did not find any association [9]. Two retrospective analyses investigated the efficacy of statin therapy to prevent KSD, and found that these lipid-lowering agents decreased the risk of developing KSD [28,29]. Despite the discrepancies in the trends of lipid profiles between different studies, their results raise the possibility of another modifiable factor to prevent KSD.

Nevertheless, these previous studies have reported inconsistent findings, the sample sizes have been limited, and there have been differences in the study population and design. Moreover, the association between lipid profile and KSD has not be examined in a large cohort follow-up study. Therefore, the aim of this longitudinal study was to investigate the association between lipid profile, including Chol/HDL-C ratio, triglycerides, low-density lipoprotein cholesterol (LDL-C), high-density lipoprotein cholesterol (HDL-C), and total cholesterol (Chol) with baseline and incident KSD in around 27,000 participants enrolled in the largest biobank in Taiwan.

## 2. Materials and Methods

### 2.1. Ethics Statement

The Institutional Review Board (IRB) of Kaohsiung Medical University Hospital approved this study (KMUHIRB-E(I)-20210058). The Ethics and Governance Council and the IRB of Biomedical Science Research, Academia Sinica, Taiwan, gave ethical approval for the Taiwan Biobank (TWB). All participants signed written informed consent before participating in the study, which followed the Declaration of Helsinki.

### 2.2. TWB

The TWB is the largest biobank in Taiwan, and it consists of genomic and lifestyle data of Taiwanese residents [30,31]. The participants in the TWB are enrolled from the community, with the inclusion criteria being: (1) age 30–70 years and (2) no cancer history. After providing written informed consent, the participants provided blood samples and underwent a physical examination. All of the participants also underwent a face-to-face interview with a TWB researcher, during which they completed a questionnaire asking about dietary habits, family medical history, personal medical history, and lifestyle factors.

### 2.3. Demographic, Laboratory, and Medical Data

Baseline data including demographics (sex and age), smoking and alcohol history, laboratory data (uric acid, LDL-C, HDL-C, triglycerides, Chol, albumin, and fasting glucose), and medical history (hypertension and diabetes mellitus [DM]) were recorded. Estimated glomerular filtration rate (eGFR) was estimated using the 4-variable Modification of Diet in Renal Disease study equation [32].

### 2.4. Presence of KSD

The participants were asked in a standardized interview whether they had ever had KSD. They were then asked again during follow-up interviews.

### 2.5. Study Participants

**Figure 1:**
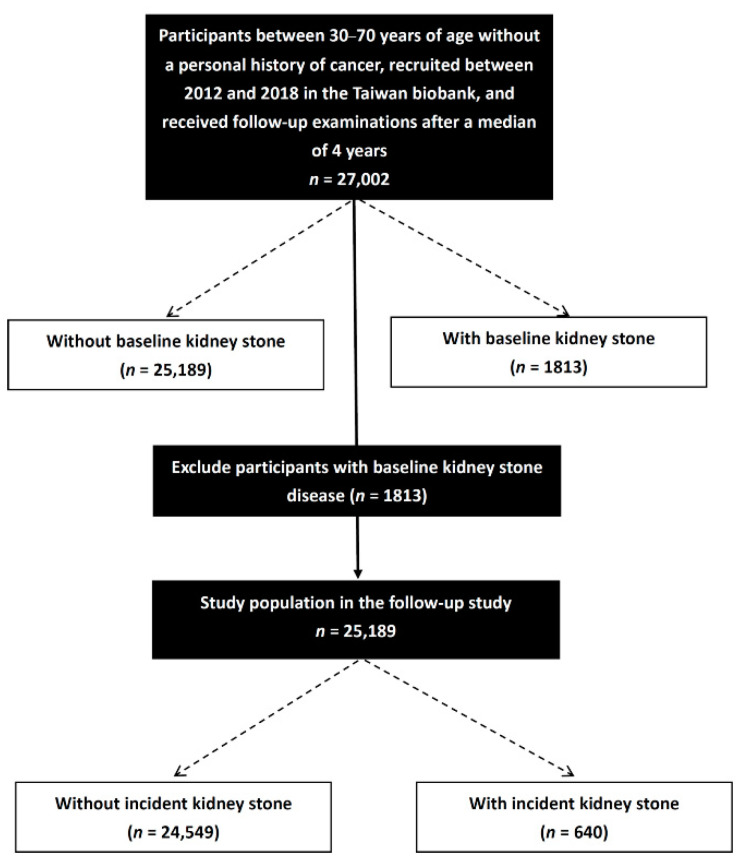
Flowchart of study population.

Overall, 27,002 participants who were followed for a median period of 4 years and had complete follow-up data were enrolled. The association between lipid profile and KSD prevalence was first evaluated in the whole cross-sectional cohort (*n* = 27,002). Participants with a KSD history (*n* = 1813) were then excluded from analysis of the longitudinal cohort. Finally, we examined the association between lipid profile and incident KSD in the participants without baseline KSD (*n* = 25,189) (Figure 1).

### 2.6. Statistical Analysis

All statistical analyses were performed using SPSS version 20.0 for Windows (SPSS Inc., Chicago, IL, USA). Data are presented as percentages or means ± standard deviations. Differences in categorical variables were analyzed using the chi-square test, and differences in continuous variables were analyzed using independent t-tests. Associations between lipid profile quartiles with baseline and incident KSD were examined using logistic regression analysis. The quartile cutoff values were: ≤66, 67–93, 94–136 and >137 mg/dL for triglycerides; ≤171, 172–193, 194–217 and >218 mg/dL for Chol; ≤45, 46–63, 54–62 and >63 mg/dL for HDL-C; ≤100, 101–120, 121–141 and >142 mg/dL for LDL-C; and ≤3.02, 3.02–3.63, 3.64–4.38 and >4.39 for Chol/HDL-C ratio. Quartiles 1 of Chol/HDL-C, Chol, triglycerides, LDL-C, and HDL-C were used as reference categories according to the lowest incidence rate. Significant variables in univariable analysis were entered into multivariable analysis. A *p* value of less than 0.05 was considered to indicate a statistically significant difference.

## 3. Results

The mean age of the 27,002 participants (9552 males and 17,450 females) was 51.2 ± 10.4 years. The participants were stratified into two groups according to baseline KSD (−) (*n* = 25,189; 93.3%) or KSD (+) (*n* = 1813; 6.7%).

### 3.1. Comparison of Clinical Characteristics among Participants According to Baseline KSD

A comparison of the clinical characteristics among the two groups is shown in Table 1. Compared to the participants without baseline KSD, those with baseline KSD were older and male predominant and had higher smoking and alcohol history, higher prevalence of DM and hypertension, higher fasting glucose, higher albumin, higher triglyceride, lower HDL-C, higher Chol/HDL-C ratio, and lower eGFR. However, total cholesterol and LDL-C levels were not significantly diffident between the two groups.

### 3.2. Associations among Lipid Profile Quartiles with Baseline KSD

Table 2 shows the association of quartile of lipid profile and baseline KSD using multivariable linear regression analysis in all study participants (*n* = 27,002). After adjusting for age, sex, smoking and alcohol history, diabetes, hypertension, fasting glucose, albumin, and eGFR (significant variables of Table 1), the participants in quartile 4 of triglyceride (vs. quartile 1; odds ratio [OR], 1.269; 95% confidence interval [CI], 1.095 to 1.470; *p* = 0.002), quartile 3 of HDL-C (vs. quartile 1; OR, 0.844; 95% CI, 0.732 to 0.972; *p* = 0.019), quartile 4 of HDL-C (vs. quartile 1; OR, 0.809; 95% CI, 0.694 to 0.943; *p* = 0.003), and quartile 4 of Chol/HDL-C ratio (vs. quartile 1; OR, 1.207; 95% CI, 1.041 to 1.400; *p* = 0.013) were significantly associated with baseline KSD.

**Figure 2:**
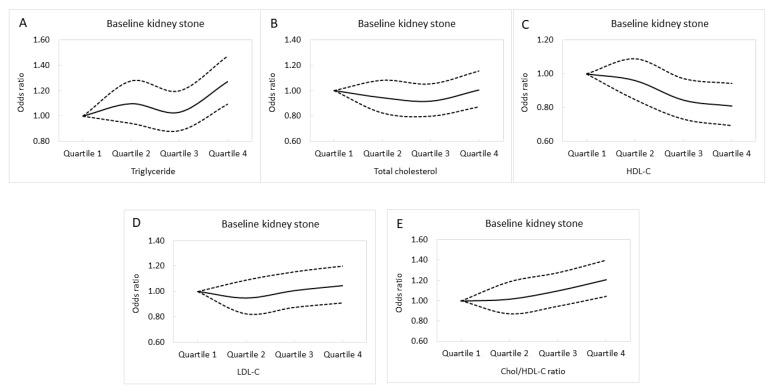
The adjusted curves of baseline kidney stone disease among the quartiles of triglyceride (**A**), total cholesterol (**B**), HDL-C (**C**), LDL-C (**D**), and Chol/HDL-C (**E**). HDL-C, high-density lipoprotein cholesterol; LDL-C, low-density lipoprotein cholesterol; Chol/HDL-C, the ratio of total cholesterol to HDL-C. Dashed lines mean odds ratio. Solid lines mean 95% confidence interval.

Figure 2A–E illustrates the adjusted curves of baseline KSD among the quartiles of lipid profile.

After exclusion the participants with a history of KSD (*n* = 1813), the mean age of study population in the follow-up study (8471 males and 16,718 females) was 51.0 ± 10.4 years. The participants were stratified into two groups according to incident KSD (−) (*n* = 24,549; 97.5%) or KSD (+) (*n* = 640; 2.5%).

### 3.3. Comparison of Clinical Characteristics among Participants According to Incident KSD

A comparison of the clinical characteristics among the two groups is shown in Table 3. Compared to the participants without incident KSD, those with incident KSD were older and male predominant and had higher smoking and alcohol history, higher prevalence of hypertension, higher fasting glucose, higher albumin, higher triglyceride, lower HDL-C, higher LDL-C, higher Chol/HDL-C ratio, and lower eGFR. However, total cholesterol was not significantly diffident between the two groups.

### 3.4. Associations among Lipid Profile Quartiles with Incident KSD

Table 4 shows the association of quartile of lipid profile and incident KSD using multivariable linear regression analysis in all study participants (*n* = 25,189). After adjusting for age, sex, smoking and alcohol history, hypertension, fasting glucose, albumin, and eGFR (significant variables of Table 3), the participants in quartile 2 of triglyceride (vs. quartile 1; OR, 1.463; 95% CI, 1.138 to 1.881; *p* = 0.003), quartile 3 of triglyceride (vs. quartile 1; OR, 1.458; 95% CI, 1.134 to 1.875; *p* = 0.003), quartile 4 of triglyceride (vs. quartile 1; OR, 1.532; 95% CI, 1.190 to 1.972; *p* = 0.001), quartile 2 of total cholesterol (vs. quartile 1; OR, 1.322; 95% CI, 1.059 to 1.651; *p* = 0.014), quartile 4 of HDL-C (vs. quartile 1; OR, 0.772; 95% CI, 0.600 to 0.993; *p* = 0.044), quartile 3 of LDL-C (vs. quartile 1; OR, 1.390; 95% CI, 1.108 to 1.743; *p* = 0.004), quartile 3 of Chol/HDL-C ratio (vs. quartile 1; OR, 1.381; 95% CI, 1.082 to 1.763; *p* = 0.010), and quartile 4 of Chol/HDL-C ratio (vs. quartile 1; OR, 1.403; 95% CI, 1.097 to 1.793; *p* = 0.007) were significantly associated with incident KSD.

**Figure 3:**
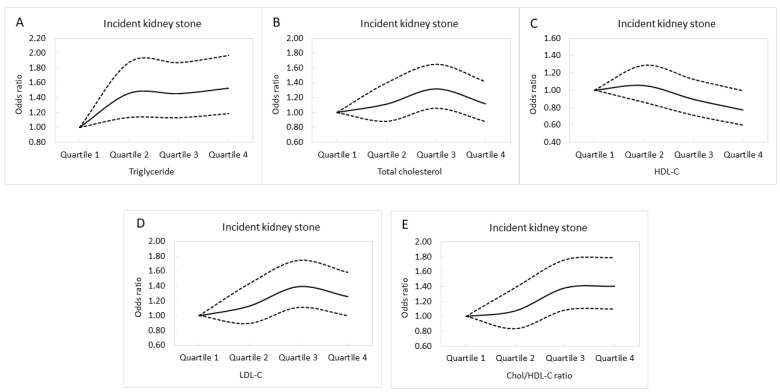
The adjusted curves of incident kidney stone disease among the quartiles of triglyceride (**A**), total cholesterol (**B**), HDL-C (**C**), LDL-C (**D**), and Chol/HDL-C (**E**). HDL-C, high-density lipoprotein cholesterol; LDL-C, low-density lipoprotein cholesterol; Chol/HDL-C, the ratio of total cholesterol to HDL-C. Dashed lines mean odds ratio. Solid lines mean 95% confidence interval.

Figure 3A–E illustrates the adjusted curves of incident KSD among the quartiles of lipid profile.

## 4. Discussion

In this longitudinal study of 27,002 participants from the TWB, we investigated the association between lipid profile with baseline and incident KSD. We found that hypertriglyceridemia, high Chol/HDL-C, and low HDL-C were associated with baseline and incident KSD.

Our results showed that the participants with hypertriglyceridemia (67–93 mg/dL) had a 1.463-fold higher risk of incident KSD, and that low HDL-C (>63 mg/dL) protected against incident KSD formation. Most of the recent studies demonstrating an association between KSD and dyslipidemia have been cross-sectional analyses [5,6,7,9,10,27,33,34] and case-control studies [11,15,35]. Three retrospective cohort studies followed the patients for more than 5 years and found that those with dyslipidemia were at a greater risk of KSD [13,14] and recurrent KSD [12]. In addition, one prospective cohort study followed the patients for 7 years and found that hypertriglyceridemia increased the risk of developing KSD [8]. Our findings are consistent with recent studies that the subjects with stone formation had higher serum triglyceride and lower serum HDL-C levels compared to those without stone formation [11,15,27,29]. On the other hand, lower serum LDL-C and total cholesterol levels have been reported in patients with stones compared to those without stones [11,15,29]. Our results are also similar to cohort studies exploring the risk of incident KSD, which have also reported associations between hypertriglyceridemia [8] and low HDL cholesterolemia [13] with a higher risk of urolithiasis. Both low HDL cholesterolemia and hypertriglyceridemia are metabolic syndrome components, and insulin resistance, obesity, and hypertension frequently coexist with these dyslipidemic conditions. Several studies have shown an association between the chronic oxidative stress and inflammation which occur in patients with metabolic syndrome, including the expression of inflammatory molecules and the early processes of kidney stone formation [36,37,38]. It is evident that lifestyle habits influence metabolic syndrome, and they have also been associated with renal stone formation. Naya et al. found that arachidonic acid in food was associated with urinary oxalate excretion [39]. Meschi et al. summarized the relevant evidence and proposed that a lower fat intake could reduce the risk of KSD [40].

Some studies have reported findings inconsistent with our study. For example, two cross-sectional analyses did not find an association between KSD and lipid profiles [5,7]. There are several possible explanations for this discrepancy. First, the study populations were different. Dyslipidemia may have different impacts in different ethnicities due to differences in genetic background, geographic environment, and lifestyle factors. Second, the sample sizes were different. The two aforementioned studies included 220 and 839 nephrolithiasis patients, respectively, compared to 1813 patients with KSD in the present study. This may have limited the power to detect the possible risk factors for dyslipidemia.

Torricelli et al. reported that serum lipid profiles also influenced urine metabolic profiles and stone composition. They reported that patients with low HDL cholesterolemia or hypertriglyceridemia had higher urinary sodium, oxalate, and uric acid excretion and lower pH [41]. Other studies have also reported similar urine findings in patients with dyslipidemia [6,12]. Liu et al. demonstrated that statin therapy could alter the urine composition in patients with KSD, with an increase in pH and citrate level, and decrease in uric acid level [42]. The abnormal urine biochemistry in patients with dyslipidemia could partially be explained by the hypothesis that insufficient HDL may compromise the HDL-related anti-inflammatory reduction in insulin resistance [13,43]. On the other hand, Bobulescu et al. found that diabetic fatty rats with high renal triglyceride content had lower urinary pH, urinary ammonium, and brush border membrane Na^+^/H^+^ exchanger-3 (NHE3), which is a major mediator of ammonium excretion. They also incubated long-chain fatty acids with kidney cells, and their results showed an association between the accumulation of intracellular lipids with a dose-dependent decrease in NHE3 activity [44]. These results suggest that decreased renal ammonium secretion could be the consequence of renal steatosis. Another plausible explanation for the association between dyslipidemia and KSD was implied in a review article by Stoller et al. [45]. They reported that a change in blood flow from laminar to turbulent at the papillary tip in the vasa recta may increase the risk of vascular injury and also that the cholesterol content in kidney stones may be caused by free cholesterol leaking from plasma in the fenestrated ascending vasa recta. This atherosclerotic-like plaque may then lead to vessel wall calcification and provide a nidus for the formation of calculi [45].

In this study, we also found that the participants with an elevated Chol/HDL-C ratio (>3.64) had a 1.381-fold increased risk off incident KSD. Dyslipidemia is highly associated with cardiovascular diseases, and many studies have examined the power of the various lipoproteins in predicting cardiovascular risk. Compared to using LDL-C alone to evaluate cardiovascular risk, which may underestimate the impact of very LDL-C, the Chol/HDL-C ratio has been shown to be a better predictor of cardiovascular risk [46,47,48]. An association between insulin resistance and a high Chol/HDL-C ratio has also been reported [46]. These findings suggest that atherosclerotic diseases may not be caused solely by a single lipoprotein, but instead by a balance between atheroprotective and atherogenic lipoproteins. Cupisti et al. investigated insulin resistance and urine composition in patients with calcium nephrolithiasis and found an association between higher insulin resistance and lower excretion of urinary citrate [49]. Moreover, Eisner et al. reported that stone formers with type 2 DM had lower urine pH and higher levels of urinary oxalate excretion than those without DM [50]. Urinary acidification has been reported to be a risk factor for uric acid stones due to low urate solubility in conditions of low urinary pH [51]. Abate et al. suggested that the mechanism underlying insulin resistance-induced KSD is probably related to induced defects in renal ammoniagenesis and increased renal citrate reabsorption [51].

To the best of our knowledge, this is the first longitudinal study to investigate associations among lipid profiles and incident KSD in Taiwan. The strengths of this study include the inclusion of a large number of participants with a long follow-up period. However, there were also several limitations. First, data on medications which may have affected dyslipidemia and KSD were not available. Second, data on the presence of KSD were obtained from self-reported questionnaires, and KSD was not verified radiographically. However, this method has been used in previous studies [6,52], and Wu and colleagues reported moderate agreement between claims record data and self-reported renal diseases in Taiwan [53]. Third, data on daily fluid intake and diet were also not available, as they fluctuate making them difficult to measure in detail. In addition, detailed data of the type of stones were also lacking. However, in Taiwan, calcium oxalate is the most common type of kidney stone, followed by calcium phosphate and uric acid, and magnesium ammonium phosphate stones are seldom found [54]. Finally, as all the participants were of Chinese ethnicity, the generalizability of our results may be limited.

## 5. Conclusions

In conclusion, our results demonstrated that hypertriglyceridemia, low HDL-C, and high Chol/HDL-C were associated with a higher risk of developing kidney stones. Our findings may imply that the optimal management of dyslipidemia can lower the risk of developing kidney stones.

## Figures and Tables

**Table 1:** Comparison of clinical characteristics among participants according to baseline kidney stone disease in all study participants (*n* = 27,002).

Characteristics	Baseline Kidney Stone (−) (*n* = 25,189)	Baseline Kidney Stone (+) (*n* = 1813)	*p*
Age (year)	51.0 ± 10.4	54.1 ± 9.2	<0.001
Male gender (%)	33.6	59.6	<0.001
Smoking history (%)	23.2	39.1	<0.001
Alcohol history (%)	8.2	11.8	<0.001
DM (%)	5.0	9.4	<0.001
Hypertension (%)	12.1	25.5	<0.001
Laboratory parameters			
Fasting glucose (mg/dL)	95.9 ± 20.0	100.3 ± 24.6	<0.001
Albumin (g/dL)	4.55 ± 0.23	4.57 ± 0.23	<0.001
Triglyceride (mg/dL)	112.9 ± 82.5	129.6 ± 90.4	<0.001
Total cholesterol (mg/dL)	195.5 ± 35.4	194.3 ± 35.5	0.150
HDL-C (mg/dL)	54.5 ± 13.2	50.6 ± 12.9	<0.001
LDL-C (mg/dL)	121.6 ± 31.6	122.4 ± 32.0	0.307
Chol/HDL-C ratio	3.76 ± 1.02	4.03 ± 1.08	<0.001
eGFR (mL/min/1.73 m^2^)	103.2 ± 23.8	96.3 ± 24.3	<0.001

Abbreviations. DM, diabetes mellitus; HDL-C, high-density lipoprotein cholesterol; LDL-C, low-density lipoprotein cholesterol; Chol/HDL-C, the ratio of total cholesterol to HDL-C; eGFR, estimated glomerular filtration rate.

**Table 2:** Association of quartile of lipid profile and baseline kidney stone disease using logistic regression analysis in all study participants (*n* = 27,002).

Quartile of Lipid Profile	Univariable (Baseline Kidney Stone)		Multivariable (Baseline Kidney Stone)	
	Odds Ratio (95% CI)	*p*	Odds Ratio (95% CI)	*p*
Triglyceride				
Quartile 1	Reference		Reference	
Quartile 2	1.328 (1.145–1.540)	<0.001	1.097 (0.943–1.276)	0.229
Quartile 3	1.416 (1.223–1.639)	<0.001	1.030 (0.885–1.198)	0.702
Quartile 4	1.988 (1.730–2.284)	<0.001	1.269 (1.095–1.470)	0.002
Total cholesterol				
Quartile 1	Reference		Reference	
Quartile 2	0.912 (0.798–1.041)	0.173	0.944 (0.823–1.082)	0.407
Quartile 3	0.885 (0.774–1.011)	0.072	0.917 (0.798–1.053)	0.220
Quartile 4	0.936 (0.819–1.069)	0.328	1.004 (0.872–1.155)	0.956
HDL-C				
Quartile 1	Reference		Reference	
Quartile 2	0.741 (0.6570.837)	<0.001	0.961 (0.848–1.089)	0.532
Quartile 3	0.549 (0.480–0.627)	<0.001	0.844 (0.732–0.972)	0.019
Quartile 4	0.452 (0.393–0.521)	<0.001	0.809 (0.694–0.943)	0.003
LDL-C				
Quartile 1	Reference		Reference	
Quartile 2	0.955 (0.833–1.094)	0.507	0.948 (0.825–1.090)	0.456
Quartile 3	1.035 (0.904–1.183)	0.621	1.006 (0.876–1.155)	0.929
Quartile 4	1.083 (0.948–1.236)	0.242	1.045 (0.911–1.200)	0.529
Chol/HDL-C ratio				
Quartile 1	Reference		Reference	
Quartile 2	1.228 (1.055–1.429)	0.008	1.018 (0.873–1.189)	0.816
Quartile 3	1.563 (1.352–1.807)	<0.001	1.099 (0.946–1.277)	0.218
Quartile 4	2.012 (1.750–2.313)	<0.001	1.207 (1.041–1.400)	0.013

Values expressed as odds ratio and 95% confidence interval (CI). Adjusted for age, sex, smoking and alcohol history, diabetes, hypertension, fasting glucose, albumin, and eGFR (significant variables of Table 1). The cutoff values of quartiles were ≤66, 67–93, 94–136 and >137 mg/dL of triglyceride; ≤171, 172–193, 194–217 and >218 mg/dL of total cholesterol; ≤45, 46–63, 54–62 and >63 mg/dL of HDL-C; ≤100, 101–120, 121–141 and >142 mg/dL of LDL-C; and ≤3.02, 3.02–3.63, 3.64–4.38 and >4.39 of Chol/HDL-C ratio. HDL-C, high-density lipoprotein cholesterol; LDL-C, low-density lipoprotein cholesterol; Chol/HDL-C, the ratio of total cholesterol to HDL-C.

**Table 3:** Comparison of clinical characteristics among participants according to incident kidney stone disease in study participants without baseline kidney stone disease (*n* = 25,189).

Characteristics	Incident Kidney Stone (−) (*n* = 24,549)	Incident Kidney Stone (+) (*n* = 640)	*p*
Age (year)	51.0 ± 10.4	52.1 ± 9.8	0.005
Male gender (%)	33.1	55.6	<0.001
Smoking history (%)	22.9	37.3	<0.001
Alcohol history (%)	8.0	13.0	<0.001
DM (%)	4.9	6.4	0.093
Hypertension (%)	12.0	18.4	<0.001
Laboratory parameters			
Fasting glucose (mg/dL)	95.9 ± 19.9	98.3 ± 20.5	0.002
Albumin (g/dL)	4.55 ± 0.23	4.58 ± 0.24	0.002
Triglyceride (mg/dL)	112.4 ± 81.5	130.7 ± 111.7	<0.001
Total cholesterol (mg/dL)	195.5 ± 35.5	196.7 ± 33.4	0.407
HDL-C (mg/dL)	54.6 ± 13.2	21.3 ± 13.2	<0.001
LDL-C (mg/dL)	121.5 ± 31.6	124.6 ± 31.4	0.013
Chol/HDL-C ratio	3.75 ± 1.02	4.01 ± 1.03	<0.001
eGFR (mL/min/1.73 m^2^)	103.3 ± 23.8	99.2 ± 23.9	<0.001

Abbreviations. DM, diabetes mellitus; HDL-C, high-density lipoprotein cholesterol; LDL-C, low-density lipoprotein cholesterol; Chol/HDL-C, the ratio of total cholesterol to HDL-C; eGFR, estimated glomerular filtration rate.

**Table 4:** Association of quartile of lipid profile and incident kidney stone disease using logistic regression analysis in study participants without baseline kidney stone disease (*n* = 25,189).

Quartile of Lipid Profile	Univariable (Incident Kidney Stone)		Multivariable (Incident Kidney Stone)	
	Odds Ratio (95% CI)	*p*	Odds Ratio (95% CI)	*p*
Triglyceride				
Quartile 1	Reference		Reference	
Quartile 2	1.636 (1.275–2.098)	<0.001	1.463 (1.138–1.881)	0.003
Quartile 3	1.765 (1.380–2.257)	<0.001	1.458 (1.134–1.875)	0.003
Quartile 4	2.091 (1.644–2.660)	<0.001	1.532 (1.190–1.972)	0.001
Total cholesterol				
Quartile 1	Reference		Reference	
Quartile 2	1.083 (0.863–1.360)	0.491	1.113 (0.885–1.399)	0.361
Quartile 3	1.271 (1.021–1.582)	0.032	1.322 (1.059–1.651)	0.014
Quartile 4	1.045 (0.830–1.315)	0.708	1.119 (0.883–1.417)	0.353
HDL-C				
Quartile 1	Reference		Reference	
Quartile 2	0.861 (0.706–1.051)	0.141	1.054 (0.860–1.291)	0.614
Quartile 3	0.638 (0.512–0.794)	<0.001	0.895 (0.712–1.126)	0.345
Quartile 4	0.493 (0.390–0.623)	<0.001	0.772 (0.600–0.993)	0.044
LDL-C				
Quartile 1	Reference		Reference	
Quartile 2	1.153 (0.913–1.456)	0.233	1.127 (0.891–1.426)	0.317
Quartile 3	1.424 (1.137–1.782)	0.002	1.390 (1.108–1.743)	0.004
Quartile 4	1.307 (1.041–1.642)	0.021	1.256 (0.997–1.583)	0.054
Chol/HDL-C ratio				
Quartile 1	Reference		Reference	
Quartile 2	1.223 (0.948–1.578)	0.122	1.075 (0.832–1.391)	0.579
Quartile 3	1.755 (1.383–2.228)	<0.001	1.381 (1.082–1.763)	0.010
Quartile 4	2.044 (1.619–2.581)	<0.001	1.403 (1.097–1.793)	0.007

Values expressed as odds ratio and 95% confidence interval (CI). Adjusted for age, sex, smoking and alcohol history, hypertension, fasting glucose, albumin and eGFR (significant variables of Table 3). The cutoff values of quartiles were ≤66, 67–93, 94–136 and >137 mg/dL of triglyceride; ≤171, 172–193,194–217 and >218 mg/dL of total cholesterol; ≤45, 46–63, 54–62 and >63 mg/dL of HDL-C; ≤100, 101–120, 121–141 and >142 mg/dL of LDL-C; and ≤3.02, 3.02–3.63, 3.64–4.38 and >4.39 of Chol/HDL-C ratio. HDL-C, high-density lipoprotein cholesterol; LDL-C, low-density lipoprotein cholesterol; Chol/HDL-C, the ratio of total cholesterol to HDL-C.

## Data Availability

The data underlying this study is from the Taiwan Biobank. Due to restrictions placed on the data by the Personal Information Protection Act of Taiwan, the minimal data set cannot be made publicly available. Data may be available upon request to interested researchers. Please send data requests to: Szu-Chia Chen. Division of Nephrology, Department of Internal Medicine, Kaohsiung Medical University Hospital, Kaohsiung Medical University.
